# Prevalence and patterns of the concurrent use of anticholinergics for the motor symptoms of Parkinson’s disease and acetylcholinesterase inhibitors in Parkinson’s disease patients with dementia: a cross-sectional study using Korea National Health Insurance claims data

**DOI:** 10.1186/s12877-022-03296-w

**Published:** 2022-07-21

**Authors:** Deborah Baik, Yun Mi Yu, Sun-Young Jung, Hye-Young Kang

**Affiliations:** 1 College of Pharmacy, Yonsei Institute of Pharmaceutical Sciences, Yonsei University, 85 Songdogwahak-ro, Incheon, 21983 South Korea; 2 College of Pharmacy, Chung-Ang University, Seoul, South Korea

**Keywords:** Acetylcholinesterase inhibitor, Cholinergic antagonist, Drug utilization, Insurance claims data, Parkinson’s disease, Dementia

## Abstract

**Background:**

The concurrent use of anticholinergics and acetylcholinesterase inhibitors (ACHEIs) in Parkinson’s disease (PD) patients with dementia should be avoided because the opposing pharmacological actions of both drugs reduce the treatment efficacy. We aimed to investigate the prevalence of the concurrent use of these two types of drugs in Korean patients.

**Methods:**

In the 2017 Health Insurance Review and Assessment Service–National Aged Patient Sample data, comprising insurance claims records for a 10% random sample of patients aged ≥ 65 years in Korea, “concurrent use” was defined as the overlapping of anticholinergic and ACHEI doses for at least 2 months.

**Results:**

Among 8,845 PD patients with dementia, 847 (9.58%) were co-administered anticholinergics, used to treat the motor symptoms of PD, and ACHEIs for a mean duration of 7.7 months. A total of 286 (33.77% of all co-administered) patients used both drug types concurrently all year. About 80% of concurrent users were prescribed each drug by the same prescriber, indicating that coadministration may not be due to a lack of information sharing between providers. Logistic regression analysis showed that patients mainly treated at clinics (odds ratio (OR), 1.541; 95% confidence interval (CI), 1.158–2.059), hospitals (OR, 2.135; 95% CI, 1.586–2.883), and general hospitals (OR, 1.568; 95% CI, 1.221–2.028) were more likely to be co-prescribed anticholinergics and ACHEIs than those mainly treated at tertiary-care hospitals. PD patients with dementia treated at healthcare organizations located in areas other than the capital city had an approximately 22% higher risk of concurrent use (OR: 1.227, 95% CI: 1.046–1.441).

**Conclusions:**

The concurrent use of anticholinergics for the motor symptoms of PD and ACHEIs in elderly Korean PD patients with dementia cannot be ignored, and strategies that mitigate potentially inappropriate concurrent drug use are required.

## Background

Parkinson’s disease (PD) is a common neurodegenerative disease [1] associated with aging [2]. Neuronal degeneration affects dopaminergic and cholinergic neurotransmission, resulting in motor symptoms and cognitive impairment [1, 3]. In patients with PD, cognitive impairment is the leading cause of the loss of independence [4]. The population of Korea is aging rapidly; consequently, the number of patients with PD has increased by 1.7 times and the total medical expenditure for the treatment of PD has increased by 3.4 times from 2010 to 2018 [5]. Thus, the healthcare burden associated with the treatment of PD continues to increase at the national level.

Nearly 80% of patients in Korea with PD experience cognitive deficits and 40% have a clinical diagnosis of dementia [6]. For the treatment of motor symptoms in patients with PD, anticholinergics or drugs that directly raise dopamine levels are used [7, 8]. The main treatments for dementia are acetylcholinesterase inhibitors (ACHEIs) or memantine [9]. Anticholinergic drugs inhibit the action of acetylcholine, whereas ACHEIs prevent its degradation; therefore, these two drug classes exhibit opposing actions. Consequently, the concurrent use of anticholinergics and ACHEIs for the treatment of PD patients with dementia has been observed to diminish the treatment efficacy in many patients [4].

Several studies have investigated the concurrent use of anticholinergics and ACHEIs. Mantri et al. [4] analysed the drug therapy of patients with dementia and PD among US Medicare beneficiaries aged ≥ 65 years. The authors reported that approximately 81.3% of patients with PD receiving ACHEIs had a history of concurrent use of anticholinergics, and 45% were treated with high-potency anticholinergics. Another study with 670 PD outpatients enrolled in a clinic registry in the US between 2012 and 2020, reported that 5.4% of the study cohort were co-prescribed cholinesterase inhibitors and medications with anticholinergic properties [10]. Bayón and Sampedro analysed the use of inappropriate treatments in 500 patients with cognitive decline [11]. They reported that the risks of prescribing anticholinesterase and memantine in patients with non-Alzheimer dementia without PD, mild cognitive impairment, or psychiatric disorders potentially exceeded the benefits of the dual prescription. The results also showed that as anticholinergics work favourably on the peripheral system, they can also induce cognitive side effects. A Korean study documenting the prevalence and treatment patterns of 1,200 PD patients with dementia from 12 hospitals reported that rivastigmine, a type of ACHEI, was the most frequently prescribed drug for treating dementia [6]; however, the study did not examine its concurrent use with anticholinergics.

The concurrent use of anticholinergics and ACHEIs among PD patients with dementia is considered potentially inappropriate. The opposing pharmacological actions of the two drugs reduce the efficacy of drug therapy; consequently, patients cannot achieve the expected clinical outcome. The resulting waste of healthcare expenditure is expected to be substantial. However, there is a lack of empirical evidence regarding the magnitude of inappropriate concurrent use at the population level and its associated factors. Therefore, we conducted this study to investigate the prevalence and characteristics of the concurrent use of anticholinergics and ACHEIs in PD patients with dementia in Korea. We also sought to identify the associated factors, with the aim of developing effective strategies for avoiding the inappropriate concurrent use of these agents in PD patients with dementia.

## Methods

### Data sources and study patients

We used the 2017 Health Insurance Review and Assessment Service–Aged Patient Sample data (2017 HIRA-APS, serial number: HIRA-APS-2017–0020), a national representative data comprising a random sample of 10% (approximately 700,000 patients) of elderly Korean patients (aged ≥ 65 years) each year. The patient samples were extracted using a stratified random sampling method from the claims data of the Korean National Health Insurance (NHI) [12]. this data is collected when healthcare providers submit a claim to HIRA for reimbursement for a service provided to patients [12]. HIRA-APS data include information on the sociodemographic characteristics of patients such as age, sex, and type of the National Health Security (NHS) program in which the patient has enrolled [i.e. contributory NHI or Medical Aid (a public assistant program)]. Moreover, in includes diagnosis and procedure codes for the NHS-covered inpatient and outpatient services provided to patients, prescription drugs, and selected characteristics of healthcare providers [12]. The research protocol was approved by the Institutional Review Board (IRB) of Yonsei University, Seoul, South Korea (IRB No. 7001988–201,910-HR-735-01E). The informed consent requirement from the study population was waived by the board on account of the retrospective nature of the study. All methods were performed in accordance with the declaration of Helsinki.

PD patients with dementia, who can be also referred to as dementia patients with PD, were defined as patients having at least one claims record with a diagnosis of PD (International Classification of Disease 10^th^ (ICD-10^th^) code: G20 [Parkinson disease] or G21 [secondary parkinsonism]) and at least one claims record with a diagnosis of dementia (ICD-10^th^ code: F00 [dementia in Alzheimer’s disease], F01 [vascular dementia], F02 [dementia in other diseases classified elsewhere], or F03 [unspecified dementia]) in 2017.

### Defining study drugs

After all the claims records of PD patients with dementia in 2017 were extracted, we collected all prescribed medication information for individual patients. We defined ‘concurrent drug use’ as a condition in which anticholinergic, used to treat the motor symptoms of PD, and ACHEI doses overlapped for at least 2 months. Cases with an overlap period shorter than 2 months were excluded from the definition of ‘concurrent use’, because we assumed that a short overlap period may have occurred during the process of switching between the two drugs. However, because the choice of a 2-month overlap period was an arbitrary cut-off point, we performed a sensitivity analysis with concurrent use defined as an overlap of at least 1 month or as an overlap from the same prescription.

Based on the guidelines of the National Institute for Health and Care Excellence (NICE) [13] and the recommendations by the Journal of the American Medical Association (JAMA) [7], trihexyphenidyl and benztropine were included in the analysis as the anticholinergic drugs used to treat the motor symptoms of PD. Based on the NICE guidelines [9], donepezil, galantamine, and rivastigmine were included as the ACHEIs used to treat dementia.

### Data analysis

The annual prevalence of the concurrent use of anticholinergics used to treat the motor symptoms of PD, and ACHEIs in 2017 among elderly Korean PD patients with dementia was calculated. To examine the patterns and potential predictors of concurrent use, various related factors were analysed. For example, analysis of the duration of concurrent use was employed as one of the methods to analyse the degree of concurrent use In addition, we investigated whether the concurrent use of the two drugs originated from the same or different prescribers.

We conducted a multivariate logistic regression analysis, with the dependent variable denoted as “1” if a patient had concurrent use and “0” otherwise. The patient and provider characteristics available in the claims records that were considered to be associated with concurrent use were included as predictors in the regression model. The patient characteristics were age, sex, type of National Health Security program enrolled (i.e., National Health Insurance or Medical Aid), and comorbidities. We sought to identify the comorbidities that were potentially associated with concurrent use. Therefore, we compiled a list of diseases reported as contraindications, warnings, and precautions in the product information of each drug included in the study [14–21]. Among the identified diseases, 37 that showed a significant difference in prevalence between the concurrent and non-concurrent use groups were used as covariates.

The characteristics of the healthcare providers who treated the PD patients with dementia included the medical specialty of the provider and the type and location of the healthcare organizations where the provider worked. For PD patients with dementia visiting more than one provider for the treatment of PD or dementia within 1 year, we defined the most-frequently visited healthcare organization as the provider. All statistical analyses were performed using SAS version 9.4 (SAS Institute, Inc., Cary, NC, USA), and the level of significance was set at 5%.

## Results

Among the 686,914 patients included in the 2017 HIRA-APS data, 17,335 had PD (2.52%), 83,542 had dementia (12.16%), and 8,845 were PD patients with dementia (1.29%). Approximately 73% of the patients were ≥ 75 years of age. There were twice as many female PD patients with dementia than male PD patients with dementia (64% vs. 36%). The proportion of PD patients with dementia enrolled in the subsidized public assistant Medical Aid (MA) program was 13.25%, which was higher than the proportion of MA beneficiaries among the general older population from the HIRA-APS data (approximately 9%). Among the PD patients with dementia, the largest proportion was treated at long-term care facilities (28.46%), followed by general hospitals (27.13%), tertiary-care hospitals (16.77%), clinics (16.72%), and hospitals (10.41%). PD patients with dementia were mainly treated by neurologists (41.37%), internal medicine physicians (15.41%), and psychiatrists (14.36%, Table 1).

**Table 1:** Patient and provider characteristics of Parkinson’s disease patients with dementia

Characteristics	No. of patients (%)
Patient characteristics	
Age, years of age	
65–69	825 (9.33)
70–74	1,552 (17.55)
≥ 75	6,468 (73.13)
Sex	
Male	3,184 (36.00)
Female	5,661 (64.00)
National Health Security program enrolled	
National Health Insurance	7,668 (86.69)
Medical Aid	1,172 (13.25)
Veterans Administration	5 (0.06)
Healthcare provider characteristics	
Type of healthcare organization that the healthcare provider worked at	
Tertiary-care hospitals	1,483 (16.77)
General hospitals	2,400 (27.13)
Hospitals	921 (10.41)
Long-term care facilities	2,517 (28.46)
Clinics	1,479 (16.72)
Others ^a^	45 (0.51)
Medical specialty of the healthcare provider	
Neurology	3,659 (41.37)
Internal medicine	1,363 (15.41)
Psychiatry	1,270 (14.36)
Family medicine	734 (8.30)
Rehabilitation medicine	480 (5.43)
Neurosurgery	425 (4.80)
Surgery	385 (4.35)
Orthopedics	166 (1.88)
Others ^a^	363 (4.10)
Location of healthcare organization that the healthcare provider worked at	
Seoul (Capital city)	1,640 (18.54)
Busan	716 (8.09)
Incheon	365 (4.13)
Daegu	496 (5.61)
Gwangju	321 (3.63)
Daejeon	374 (4.23)
Ulsan	141 (1.59)
Gyeonggi	1,630 (18.43)
Gangwon	278 (3.14)
Chungcheong/Sejong	601 (6.79)
Jeonla/Jeju	1,171 (13.24)
Gyeongsang	1,112 (12.57)

*n* = 8,845 Parkinson’s disease patients with dementia

^a^ Type of healthcare organization and medical specialty of the healthcare provider, of which the proportion is < 1%, are classified as ‘Others.’

**Fig. 1:**
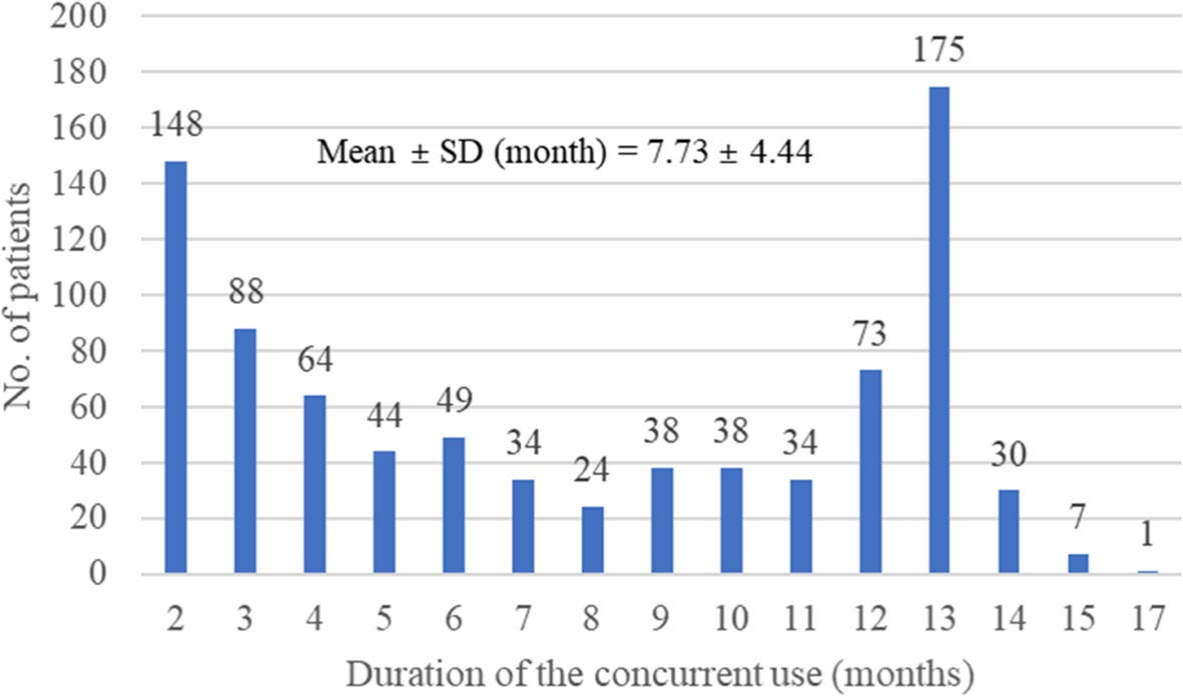
Distribution of the duration of concurrent use of anticholinergics used to treat the motor symptoms of Parkinson’s disease and ACHEIs in Parkinson’s disease patients with dementia. *ACHEI, acetylcholinesterase inhibitor

Approximately 19.20% of the PD patients with dementia were prescribed anticholinergics to treat their motor symptoms. ACHEIs were predominantly used to treat dementia in PD patients with dementia (92.63%). The prevalence of the concurrent use of anticholinergics for the motor symptoms of PD and ACHEIs in the PD patients with dementia was 9.58% (847 of 8,845 PD patients with dementia, Table 2). When concurrent use was defined as overlapping for at least 1 month instead of at least 2 months, the prevalence increased to 10.66%. The proportion of PD patients with dementia receiving anticholinergics for the motor symptoms of PD and ACHEIs concurrently from the same prescription was 7.93%. The mean duration of concurrent use was 7.7 months (Fig. 1), with a distribution ranging from a minimum of 2 months to a maximum of 17 months. There were 286 patients with an overlap period lasting ≥ 12 months, which accounted for 33.77% of the total concurrent use group.

**Table 2:** Analysis of drug use in Parkinson’s disease patients with dementia

Drug use modality	No. of patients (%)
Use of anticholinergics for motor symptoms in Parkinson’s disease	1,268 (19.20)
Use of ACHEIs for dementia	6,460 (92.63)
Concurrent use of anticholinergics for the motor symptoms of Parkinson’s disease and ACHEIs	
Concurrent use for ≥ 2 months	847 (9.58)
Concurrent use for ≥ 1 month	943 (10.66)
Co-prescribed in the same prescription	701 (7.93)

*n* = 8,845 Parkinson’s disease patients with dementia

*Abbreviations: ACHEI* Acetylcholinesterase inhibitor

 Anticholinergics for the motor symptoms of PD and ACHEIs were co-administered more often within the same healthcare organization (709 patients [83.71%]) than between different organizations (217 patients [25.62%], Table 3). Some of the PD patients with dementia received cotreatment from both the same and different organizations; therefore, the sum of the proportion of the two groups was greater than 100%. Approximately 80% (676/847) of the prescriptions in the concurrent use group were prescribed by the same physician. The medications were co-administered within the same organization most often in general hospitals (33.65%), followed by clinics (19.83%), hospitals (17.12%), tertiary-care hospitals (12.51%), and then long-term care hospitals (7.20%).

**Table 3:** Distribution of Parkinson’s disease patients with dementia prescribed anticholinergics used to treat the motor symptoms of Parkinson’s disease and acetylcholinesterase inhibitors (ACHEIs) concurrently^a^ by the same or different healthcare organizations

Prescribed within the same or different healthcare organizations	No. of patients who used agents concurrently ^b^ (%)
Prescribed within the same organization	709 (83.71)
Prescribed by the same or different prescribers	
Same prescriber	676 (95.35)
Different prescribers	81 (11.42)
Types of healthcare organizations	
General hospitals	285 (33.65)
Clinics	168 (19.83)
Hospitals	145 (17.12)
Tertiary-care hospitals	106 (12.51)
Long-term care hospitals	61 (7.20)
Others ^c^	6 (0.71)
Prescribed in different organizations	217 (25.62)

*n* = 847 Parkinson’s disease patients with dementia (concurrent use group)

*Abbreviations: ACHEI* Acetylcholinesterase inhibitor

^a^ Patients with concurrent use refer to patients prescribed both anticholinergics for the motor symptoms of Parkinson’s disease and ACHEIs for at least 2 months

^b^ Patients can be classified into more than one group. Thus, the sum of the proportion is greater than 100%

^c^ Type of healthcare organizations of which the proportion is < 1% are classified as “Others.”

The multivariate logistic regression analysis (Table 4) showed a decrease in concurrent use with an increase in age (odds ratio [OR]: 1.344, 95% confidence interval [CI]: 1.056–1.698 for those between 65 and 69 vs. ≥ 75 years of age). Compared with PD patients with dementia treated at tertiary-care hospitals, those treated at hospitals (OR: 2.135, 95% CI: 1.586–2.883), general hospitals (OR: 1.568, 95% CI: 1.221–2.028), and clinics (OR: 1.541, 95% CI: (1.158–2.059) had a significantly higher risk of concurrent use. Among the patients, those who mainly visited psychiatrists in 2017 were the only ones with significantly higher simultaneous prescription rates of the two drugs than patients who mainly visited neurologists in 2017 (OR: 1.898, 95% CI: 1.530–2.354). PD patients with dementia treated at healthcare organizations located in areas other than the capital city had an approximately 22% higher risk of concurrent use (OR: 1.227, 95% CI: 1.046–1.441).

**Table 4:** Logistic regression analysis of factors associated with the concurrent use of anticholinergics used to treat the motor symptoms of Parkinson’s disease and acetylcholinesterase inhibitors in Parkinson’s disease patients with dementia

Variables	No. of patients in the concurrent use group (%)	Unadjusted odds ratio (95% CI)	Logistic regression analysis results
			Adjusted odds ratio (95% CI)
Age, years			
65–69	113 (13.70)	1.649 (1.322–2.039)	1.344 (1.056–1.698)
70–74	166 (10.70)	1.244 (1.034–1.490)	1.083 (0.889–1.314)
≥ 75 (ref)	568 (8.78)	-	-
Sex			
Male (ref)	279 (8.76)	-	-
Female	568 (10.03)	1.161 (1.000–1.351)	1.019 (0.842–1.238)
National Health Security program enrolled			
NHI (ref)	711 (9.27)	-	-
MA	135 (11.52)	1.274 (1.044–1.543)	1.130 (0.912–1.392)
VA	1 (20.00)	2.447 (0.121–16.562)	1.526 (0.071–12.176)
Type of health care organization that the healthcare provider worked at			
Tertiary-care hospitals (ref)	98 (6.61)	-	-
General hospitals	271 (11.29)	1.799 (1.419–2.298)	1.568 (1.221–2.028)
Hospitals	151 (16.40)	2.772 (2.121–3.636)	2.135 (1.586–2.883)
Long-term care facilities	104 (4.13)	0.609 (0.459–0.810)	0.720 (0.516–1.004)
Clinics	216 (14.60)	2.417 (1.887–3.116)	1.541 (1.158–2.059)
Others	7 (15.56)	2.603 (1.042–5.641)	3.147 (1.211–7.206)
Medical specialty of healthcare provider			
Neurology (ref)	314 (8.58)	-	-
Internal medicine	102 (7.48)	0.862 (0.68–1.083)	0.862 (0.662–1.115)
Psychiatry	276 (21.73)	2.958 (2.479–3.529)	1.898 (1.530–2.354)
Surgery	21 (5.45)	0.615 (0.379–0.945)	0.897 (0.532–1.443)
Orthopedics	8 (4.82)	0.539 (0.241–1.038)	0.602 (0.264–1.193)
Neurosurgery	41 (9.65)	1.137 (0.797–1.583)	1.187 (0.823–1.674)
Rehabilitation medicine	20 (4.17)	0.463 (0.283–0.716)	0.677 (0.405–1.072)
Family medicine	39 (5.31)	0.598 (0.418–0.832)	0.923 (0.620–1.344)
Others	26 (7.16)	0.822 (0.53–1.221)	0.805 (0.500–1.249)
Location of healthcare organization that the healthcare provider worked at			
Capital city area (ref) ^a^	323 (8.89)	-	-
Other regions	524 (10.06)	1.147 (0.992–1.327)	1.227 (1.046–1.441)
Comorbidity ^b^			
Schizophrenia (F20)	167 (25.04)	3.683 (3.033–4.454)	2.342 (1.885–2.898)
Abnormal results of kidney function studies (R944)	19 (20.88)	2.096 (1.215–3.424)	2.227 (1.233–3.827)
Other specified forms of tremor (G252)	21 (18.26)	2.264 (1.308–3.713)	2.041 (1.135–3.493)
Bipolar affective disorder (F31)	44 (14.72)	2.240 (1.924–2.604)	1.769 (1.495–2.089)
Unspecified nonorganic psychosis (F29)	59 (23.51)	3.046 (2.236–4.086)	1.736 (1.246–2.389)
Sequelae of other and unspecified cerebrovascular diseases (I698)	300 (16.02)	1.527 (1.066–2.132)	1.604 (1.097–2.290)
Palpitations (R002)	38 (13.29)	1.915 (1.342–2.668)	1.521 (1.038–2.180)
Tremor, unspecified (R251)	49 (16.61)	1.734 (1.403–2.128)	1.502 (1.195–1.876)
Other specified postsurgical states (Z988)	39 (13.73)	1.522 (1.124–2.024)	1.460 (1.057–1.984)
Cerebral infarction, unspecified (I639)	120 (14.72)	0.693 (0.566–0.843)	0.799 (0.645–0.984)
Heart failure, unspecified (I509)	41 (16.53)	0.724 (0.549–0.938)	0.754 (0.562–0.995)
Urinary tract infection, site not specified (N390)	55 (13.61)	0.754 (0.609–0.925)	0.740 (0.586–0.927)
Hyperplasia of prostate without complication (N400)	89 (12.50)	0.739 (0.594–0.909)	0.699 (0.534–0.913)
Unstable angina (I200)	123 (7.25)	0.561 (0.335–0.881)	0.516 (0.299–0.838)
Cerebral infarction due to thrombosis of cerebral arteries (I633)	110 (7.68)	0.293 (0.104–0.643)	0.388 (0.136–0.868)
Normal-pressure hydrocephalus (G912)	106 (7.55)	0.292 (0.089–0.694)	0.358 (0.108–0.871)
Stroke, not specified as haemorrhage or infarction (I64)	78 (7.62)	0.317 (0.134–0.625)	0.345 (0.144–0.698)
Chronic kidney disease, stage 3 (N183)	22 (6.43)	0.244 (0.060–0.647)	0.217 (0.051–0.614)

*n* = 8,845 Parkinson’s disease patients with dementia

*Abbreviations: CI* Confidence interval, *NHI* National Health Insurance, *MA* Medical Aid, *VA* Veterans Administration

^a^ Capital city area includes the capital city (i.e., Seoul) and its neighboring regions (i.e., Incheon and Gyeonggi)

^b^ Of the 37 comorbid conditions with significantly different prevalence between the concurrent and non-concurrent medication groups, only 18 conditions that were significantly associated with concurrent use from the multivariate analysis are listed. ICD-10^th^ diagnosis codes are presented in parentheses

Among the comorbidities of the PD patients with dementia, some neuropsychiatric diseases, including schizophrenia (OR: 2.342, 95% CI: 1.885–2.898), psychosis (OR: 1.736, 95% CI: 1.246–2.389), and bipolar affective disorder (OR: 1.769, 95% CI: 1.495–2.089), were positively associated with concurrent use. A negative association with concurrent use was shown for urinary diseases (e.g., urinary tract infection [OR: 0.740, 95% CI: 0.586–0.927], hyperplasia of the prostate [OR: 0.699, 95% CI: 0.534–0.913], and chronic kidney disease [OR: 0.217, 95% CI: 0.051–0.614]), and cardiovascular diseases (e.g., stroke [OR: 0.345, 95% CI: 0.144–0.698], cerebral infarction [OR: 0.388, 95% CI: 0.136–0.868], and unstable angina [OR: 0.516, 95% CI: 0.299–0.838]).

## Discussion

The purpose of this study was to analyse the current status of the concurrent use of anticholinergics for the motor symptoms of PD and ACHEIs among elderly Korean PD patients with dementia. Of the 8,845 PD patients with dementia identified from the 2017 HIRA-APS data in Korea, 847 (9.58%) and 943 (10.66%) were co-administered anticholinergics for the motor symptoms of PD and ACHEIs, depending on how the concurrent use was defined. This result indicated that approximately 1 in 10 PD patients with dementia in Korea had potentially used inappropriate medication.

Our estimated prevalence of the concurrent use was much lower than that of US Medicare beneficiaries aged ≥ 65 years. Among this population, 81.3% of patients with PD receiving ACHEIs had a history of concurrent use of anticholinergics in 2014 [4]. The difference in the prevalence of concurrent use between the two studies may be partly explained by the difference in the lists of drugs included in the study. In the study in the US, a broader range of drugs was defined as anticholinergics, including those not used to treat PD, whereas our study was restricted to only anticholinergics used to treat the motor symptoms of PD.

Our analysis showed that the mean duration of concurrent use was 7.7 months. It is noteworthy that 286 patients, accounting for 33.77% of the total concurrent use group, were co-administered both drugs throughout the year. These results suggest that the extent of concurrent use among PD patients with dementia in Korea appears to be substantial.

Approximately 80% (676/847) of the patients in the concurrent use group received prescriptions from the same physician. In other words, the same physician prescribed these two drugs to the same patients for at least 2 months. This finding suggests that a lack of information sharing regarding drug therapy prescribed to patients between different healthcare providers may not be the main reason for the concurrent use of the two drugs.

The patient and provider characteristics identified in the multivariate regression analysis as being significantly associated with the concurrent use of the two drugs can provide an explanation for such behavior and potential target areas for corrective action. Concurrent use tended to decrease with age. We believe that this was probably because older patients were less likely to use anticholinergics owing to the known risks of their effects. According to the 2019 Beers [22] Criteria, a significant number of anticholinergics were classified as “potentially inappropriate medication in older adults.” The rationale for this classification of anticholinergics is the reduced clearance with advanced age; tolerance developed when used as a hypnotic; and risk of confusion, dry mouth, constipation, and other anticholinergic effects or toxicity. The proportion of PD patients with dementia enrolled in the subsidized public assistant MA program was higher than the proportion of MA beneficiaries among the general older population from the HIRA-APS data. This implies that the economic status of PD patients with dementia is lower than that of the general older population.

Compared with PD patients with dementia treated at tertiary-care hospitals, those treated at general hospitals, hospitals, or clinics had a higher chance of concurrent use. In Korea, the health law that distinguishes tertiary-care hospitals from general hospitals mainly focuses on the size of the hospital and the complexity of the healthcare services provided. It is classified according to the required specialty types and the number of beds in each hospital. Therefore, quality improvement activities, including drug utilization review, are more actively performed at tertiary-care hospitals than at the other types of healthcare organizations. Thus, the higher occurrence of inappropriate concurrent use among those types of healthcare organizations is a predictable finding.

Patients who mainly visited psychiatrists were the only patient group with a significantly higher rate of concurrent prescription of the two drugs than patients who mainly visited neurologists, who are the most common treatment providers for PD patients with dementia in Korea. In addition, patients who mainly visited healthcare organisations located outside of the capital city areas showed a significantly higher rate of concurrent prescription of the two drugs than patients who mainly visited healthcare organisations in the capital city. These findings suggest that educational programs need to target psychiatrists and clinicians working in non-capital city areas to improve prescribing behavior when treating PD patients with dementia.

Concurrent use was significantly associated with comorbidities in PD patients with dementia. A higher probability of concurrent use was observed in patients with neuropsychiatric disorders. Antipsychotic drugs are used to treat psychiatric disorders, such as schizophrenia, psychosis, and bipolar affective disorders. Antipsychotic drug-induced extrapyramidal adverse effects are very common among antipsychotic drug users [23] and can be treated with anticholinergics or amantadine [24]. Among the 847 patients, 581 in the concurrent use group were prescribed more than one prescription with the antipsychotic drugs. Thus, in many cases, the high rate of concurrent use in patients with psychiatric disorders may be the result of an inevitable situation. In addition, patients with tremor-dominant PD can use anticholinergics even when they are ≥ 60 years old [7]. Therefore, this fact has possibly contributed to increasing the prevalence of concurrent use in patients with “unspecified tremor.” Consequently, the concurrent use of these agents in patients with neuropsychiatric disorders may be unavoidable. Conversely, diseases other than neuropsychiatric disorders that increase concurrent use may serve as factors that increase inappropriate concurrent use; thus, careful monitoring of patients with these diseases is necessary.

In contrast, PD patients with dementia and comorbidities such as urinary and cardiovascular diseases were less likely to concurrently use these agents. Anticholinergics can lead to urine retention and, therefore, should be avoided in the treatment of urinary diseases such as urinary tract infection and hyperplasia of the prostate [25]. This would contribute to reducing the prevalence of the concurrent use of these agents in patients with urinary diseases [25]. Vascular dementia may occur after cardiovascular events such as stroke or cerebral infarction; therefore, anticholinergics that inhibit the action of acetylcholine should be avoided in susceptible patients [26]. This may reduce the prevalence of concurrent use in patients with cardiovascular diseases.

Our study had several limitations that should be addressed in future studies. First, we investigated only two anticholinergic drugs used to treat the motor symptoms of PD. Other anticholinergics may have been used as symptomatic treatments for other comorbidities in PD patients, accounting for the potential inappropriate concurrent use. Therefore, our analysis was limited in that it could not comprehensively examine the concurrent use of anticholinergics and ACHEIs in patients with PD and dementia. Future studies expanding the scope of this study by including other types of anticholinergics may indicate that the extent of the concurrent use with ACHEI is greater than that indicated by our findings. Second, we used data from a single year for our analysis. To investigate factors associated with concurrent use, it would be helpful to evaluate the possibility of certain conditions that trigger concurrent use. Where anticholinergics and ACHEIs are prescribed by different healthcare providers, it would be helpful to identify the providers who provided the latter prescription, identify the responsible party, and effectively target areas requiring further training. However, as the data used in this study included information for only one year, we were not able to conduct such an analysis. Furthermore, left or right censoring could have occurred because the data included information only from January to December 2017. For example, the concurrent use between the end of 2016 and the beginning of 2017 or between the end of 2017 and the beginning of 2018 could not be counted, and it could have led an underestimation in the prevalence of concurrent use.

Third, our study subjects were identified based on diagnosis codes included in the claims records. However, owing to the administrative nature of claims data, some of the diagnosis codes from the claims records may not be accurate, probably because of random coding errors or upcoding behaviours. This factor might have caused misclassification of the study subjects.

Fourth, owing to the lack of clinical information on the administrative claims data, there is a possibility that the concurrent use was misclassified. For example, patients concurrently prescribed medications inappropriately may have stopped taking them within 1 month owing to rapid recovery. In this case, these patients were excluded from the concurrent use group because we defined concurrent use as doses of the two drugs that overlapped for at least 2 months. This would result in an underestimation of the concurrent prescription rate.

Finally, our regression model might have had an omitted variable bias. For example, the intake of certain drugs may lead to the prescription of additional anticholinergics or ACHEIs. Clinicians would more frequently prescribe anticholinergics in patients who are insufficiently controlled with other PD drugs. However, due to the limitation of one-year claims data, which depend on limited clinical information and do not provide a history of treatment outcomes of PD or dementia, we were unable to include as many covariates as our hypothesis suggested.

## Conclusions

This study showed that the prevalence of concurrent anticholinergic, used to treat the motor symptoms of PD, and ACHEI drug use was approximately 10% in older PD patients with dementia in Korea. The multivariate analysis showed that younger PD patients with dementia with neuropsychiatric disease as a comorbidity, who were being treated at healthcare organizations—general hospitals, hospitals, or clinics—and were receiving treatment from psychiatry specialists or from providers outside of the capital city areas were vulnerable to the use of concurrent medication. Therefore, we suggest an intervention for this target population of PD patients with dementia to be established, if concurrent use is an avoidable situation, and to provide effective treatment and minimise the potentially inappropriate concurrent use of anticholinergics and ACHEIs.

## Data Availability

Authors used the 2017 Health Insurance Review and Assessment Service-National Aged Patient Same (HIRA-APS) data for this study and do not have permission to share the data. If there are any questions about the data in this study, please contact the corresponding author. Future researchers can request access for this data from the Health Insurance Review and Assessment Service (http://opendata.hira.or.kr).

## Abbreviations

ACHEIs: Acetylcholinesterase inhibitor
APS: Aged Patient Sample
HIRA: Health Insurance Review and Assessment Service
ICD-10th code: International classification of Disease and Related Health Problems,10th Revision
JAMA: The Journal of the American Medical Association
MA: Medical Aid
NICE: National Institute for Health and Care Excellence
PD: Parkinson disease
